# Clinical Education: Consultation-Liaison Psychology Services

**DOI:** 10.1007/s10880-025-10067-3

**Published:** 2025-02-22

**Authors:** Joanna S. Yost, Casey E. Cavanagh

**Affiliations:** University of Virginia School of Medicine, Charlottesville, USA

**Keywords:** Consultation-liaison psychology, Clinical education, Behavior management, Program development

## Abstract

Consultation-liaison (CL) psychology is a subspecialty of clinical health psychology primarily focused on providing both consultation and liaison services in the medical inpatient setting to benefit patients, families, and/or healthcare providers. The goals of this paper are threefold. The first is to educate clinicians about the scope and functions of traditional CL psychology services. The second is to identify contemporary areas of growth and development in CL psychology services. The third is to discuss practical recommendations and lessons we have learned throughout the process of establishing and sustaining CL psychology services at our organization. Additionally, we discuss the future of CL psychology particularly with regard to the dearth of CL psychology research.

## Introduction

Consultation-liaison (CL) psychology is a growing subspecialty of clinical health psychology and has been for over 40 years (e.g., Schlebusch, 1983; Wellisch & Pasnau, 1979). The primary purpose of CL psychology services is to identify and intervene on biopsychosocial factors that affect a patient’s medical condition or engagement in medical treatment. In consultation, a psychologist provides an expert opinion on diagnosis, intervention, and/or management of a patient’s mental health or care-interfering behaviors. This process involves three primary parties, the psychologist, the primary team or team requesting the consult, and the patient (Grover & Naskar, 2023; Lipowski, 1967, 1983). The liaison part of CL psychology focuses on effective communication and collaboration between patients and the medical teams and communication between the psychologist and medical teams (Grover & Naskar, 2023; Lipowski, 1967, 1983). Data on the number of psychologists providing CL services are limited. A recent study of CL psychologists indicated that 47% of CL psychologists provided services on a psychologist-led CL psychology service rather than an interdisciplinary service such as a CL psychiatry service (LaGrotte et al., 2024). CL psychology services can differ from CL psychiatry services in several ways, but perhaps most importantly they differ in the primary strategies used to intervene on the patient or system in order to address the consultation question. CL psychologists typically focus on biopsychosocial and behavioral aspects of a patient’s care and utilize evidence-based assessment, psychotherapeutic interventions, and behavioral interventions. CL psychiatrists typically focus on addressing psychiatric symptoms through the use of medication management, occasionally in conjunction with therapy. We developed and serve as co-directors of an independent CL psychology service at the University of Virginia Medical Center. Our CL psychology service has benefitted from close collaboration with our CL psychiatry service and this relationship has allowed us to provide the most comprehensive and effective care to patients, their families, and healthcare teams. The purpose of this clinical education paper is to provide education to clinicians on CL psychology services, identify contemporary issues impacting CL psychology services, and summarize practical considerations in the development and implementation of CL psychology services. 

## Models and Services of CL Psychology

While consultation and liaison roles are common across CL psychology services, the models through which these primary roles are accomplished can vary significantly. Most commonly, the differences in models relate to the degree of integration vs. independence from other services. CL psychology services can be integrated within a CL psychiatry team, specific medical services (e.g., transplant), or specific units (e.g., trauma/surgical units), or consulted for specific presentations (e.g., behavioral disturbances). Alternatively, CL psychology services may be focused on specific populations, such as pediatrics vs. adult, or may offer services throughout a hospital regardless of population or unit. Finally, a CL psychology service can exist independently or as an additional service to CL psychiatry services. Research examining the models and structure of CL psychology services is limited. Additional differences relate to the source of the consult, patient or primary team requested, and how the service is initiated, either proactively or reactively (Grover & Naskar, 2023).

## Traditional Services Provided by CL Psychologists

The psychological services traditionally provided by CL psychologists vary depending on the model and identified purpose and scope of the CL service. Broadly, CL psychologists provide evidence-based assessment and intervention to the patient, family, and/or medical team. Although research is limited, CL services improve patient satisfaction (Bullock et al., 2022) and patient-reported outcomes, such as depression and anxiety symptoms (Paterson et al., 2018). Healthcare leaders and institutions also benefit from CL psychology services, most critically through cost savings (De Araujo Andreoli et al., 2003), and reductions in length of stay (Aoki et al., 2004; Muskin et al., 2016). Further, CL psychology services have been shown to reduce the occurrence of behavioral emergencies (Yost et al., 2021). Although there are clear benefits to CL services, there is dearth of research examining the specific effects of CL psychology services on clinical and patient-reported outcomes.

### Assessment

Assessment in CL psychology is typically targeted and focused on specific behaviors and symptoms that are relevant to the consultation question and inform intervention. CL psychologists frequently use patient-reported screening and symptom assessment instruments (LaGrotte et al., 2024). CL psychologists also use behavioral observations to identify the function of disruptive or care-interfering behaviors, which in turn inform recommendations for management of these behaviors. Behavioral observations can also be taught to and conducted by frontline staff to assist CL psychologists in monitoring patient progress. More comprehensive evaluations may be conducted, but typically such services are limited to specific populations and consulting questions. For example, CL psychologists may provide pre-transplantation evaluations to patients who are admitted to the medical hospital in the absence of a dedicated transplant psychology service. As previously discussed, the assessments administered should clearly answer the consultation question or inform the intervention services to be provided.

### Individual Intervention

The extant CL psychology literature suggests that brief evidence-based psychological interventions can be effectively delivered with medical populations and in a variety of medical settings, including in the emergency department, acute care units, and intensive care units. Interventions in these settings typically focus on coping with hospitalization, adjustment to new diagnoses or change in physical functioning, coping with pain, managing psychological symptoms that may be distressing or interfering with treatment, or as a warm handoff to connect patients with outpatient psychological services (LaGrotte et al., 2024). The interventions most frequently used on CL psychology services include cognitive behavioral therapy, motivational interviewing, and acceptance commitment therapy (LaGrotte et al., 2024).

## Contemporary Services Provided by CL Psychologists

The growth and sustainability of any hospital-based service, CL psychology included, hinges on the ability to provide, expand, and develop services that are meaningful and relevant in the ever-evolving landscape of healthcare. To be successfully sustained, these services must demonstrate and maintain alignment with the organization’s needs and missions. The ability of CL psychologists to quickly and thoughtfully pivot to help address the rapidly changing needs of healthcare institutions has proven particularly useful over the past several years and provides insight into how CL psychologists can continue to develop and grow CL psychology services through problem-solving and innovation. One example is the impressive work done by CL psychologists across the US during the COVID-19 pandemic. CL psychologists developed creative methods to continue to provide intervention services to patients and family members, often utilizing new technology, during the pandemic. Additionally, many CL psychologists recognized the incredible burden that our medical colleagues were experiencing and expanded their services to provide various types of psychological support to frontline staff. Similarly, our CL psychology service and others have recognized the rapidly emerging importance of the role CL psychologists can play in the prevention and management of workplace violence (WPV).

### Behavior Management

The alarming increase in violence against healthcare workers committed by patients has been recognized as a critical area of intervention for healthcare systems. In recognition of this growing problem and concern for the safety of those providing healthcare services, the Joint Commission created new accreditation standards in 2022 that mandate healthcare organizations to implement effective WPV prevention programs. Behaviors categorized as WPV include verbal and physical aggression, threatening behavior, sexually inappropriate behavior, and property destruction. These problematic behaviors have historically been addressed in healthcare settings through security and risk management practices. However, research and practice has called for more patient-centered approaches to address incidents of WPV, which are often classified as behavioral emergencies in the medical inpatient setting (Parker et al., 2020). CL psychologists’ expertise in behavioral principles and behavior management is critical for effectively addressing these issues.

Patient behavior management provided by CL psychology services has been described in the literature (Yost et al., 2021). We, along with some of our CL psychology colleagues, have conceptualized this work similarly through a public health prevention model that includes primary, secondary, and tertiary prevention and intervention strategies (Cavanagh et al., 2024; Yost et al., 2021). Primary prevention strategies include staff training on basic principles of behavior management and effective communication as well as orientation to the CL psychology service. Innovative ways to deliver staff training for medical providers could include a combination of didactics, role-playing activities with specific skills checks and feedback, simulation training, and virtual reality-based training. Secondary prevention strategies include general behavior management plans for patients who have demonstrated minor disruptive behavior (e.g., verbal aggression such as cursing without threat) which has not escalated to interfering with care and has not resulted in a behavioral emergency. Secondary prevention strategies implemented by CL psychologists often involve a stepwise algorithm that is applied consistently to all patients who are engaging in disruptive behaviors. These algorithms provide staff with clear guidelines for consistent management based on behavioral principles that are applied across a patient’s care continuum. Tertiary prevention strategies implemented by CL psychologists involve assessing the function of a disruptive or care-interfering behavior to develop an individualized behavior management plan. To accomplish this, CL psychologists often complete direct observation of the disruptive behavior and collaborate with medical teams and frontline staff.

Effective individualized behavior management plans can only be created with both input and buy-in from all members of the care team. Multidisciplinary complex care meetings include identifying target behaviors for intervention, identifying specific reinforcements that are salient to the patient and who will provide the reinforcer, identifying a reinforcement schedule that will be used to reward engagement in appropriate behaviors and/or absence of target behaviors, problem-solving consistent application of the plan by all staff, and creating consistent standard responses to decrease inadvertent reinforcement of the maladaptive behavior.

## Practical Considerations in CL Psychology

### Developing a CL Psychology Service

CL psychology services, like other CL services, have difficulty demonstrating value through traditional productivity measures such as billing/collections or RVUs (De Araujo Andreoli et al., 2003). As such, when developing a CL psychology service, it is critical to ensure that services are designed to align with the needs of stakeholders, including institutional leadership, clinicians and other frontline staff, and patients. Understanding and addressing the stakeholder needs has been critical to the sustained success of our CL psychology service. It is critically important that the outcomes of interest be clearly defined, objective, and measurable in order to demonstrate the value and unique impact of the CL psychology service. This allows CL psychologists to track performance on each outcome, evaluate effectiveness of the service based on identified outcomes of interest, and identify areas of change and improvement.

### Value to Institutional Leaders

Often, institutional leaders are interested in targeting outcomes related to quality assurance standards set by national regulatory agencies such as Centers for Medicare and Medicaid Services (CMS) and the Joint Commission for Accreditation of Hospital Organizations-Ambulatory Care as well as financial metrics. With our CL psychology service, the primary concern for institutional leaders was the management of care-interfering behaviors and reduction in workplace violence and staff injuries. As such, the initial purpose of the service was to develop a tiered approach to behavior management to address care-interfering and disruptive behaviors. A cornerstone of the development and implementation of our service has been delivering staff trainings that teach general behavior management strategies at the primary prevention level. Our service demonstrated an increase in staff confidence in effectively utilizing behavior management strategies and a reduction in occurrence of behavioral emergencies (Yost et al., 2021). After demonstrating the value of our CL psychology service, our institution provided financial support for these efforts, which enabled us to expand the team and services we deliver.

### Value to Collaborators

Collaboration is a key concept for any CL psychology service and is another way to demonstrate value added to the institution. CL psychologists collaborate with a wide variety of staff working in medical centers, including medical teams, nursing staff, allied health professionals, ethics, patient safety/risk management, and security and/or police. These collaborations are essential in implementing effective behavior management plans as well as in building support and champions for the service. To help build these collaborations, it is critical for CL psychologists to use effective communication strategies and set clear expectations regarding services. For example, consulting teams and other clinicians should understand the CL psychology service’s coverage, anticipated time to respond to new consults, and anticipated time to provide a service. CL psychologists must also utilize effective communication strategies to convey recommendations following the completion of an initial consultation. Additionally, it is helpful for CL psychologists to regularly request both formal and informal feedback from the various staff they consult with in order to address any frustration or problems that arise with the service. Measures of staff satisfaction can be helpful in ascertaining perceived benefits as well as areas of potential modification and growth of the service. Additionally, measures that evaluate improvement in staff skill or staff comfort in managing care-interfering patient behaviors can demonstrate the benefit of interventions such as informal coaching sessions with staff or formal staff training lead by CL psychologists.

### Value to Patients

Patient outcomes on key performance indicators as well as patient satisfaction measures are additional measures that CL psychology services can utilize to demonstrate value. A previous study of a CL psychology service in a single acute care hospital found that 83% of patients rated the service as helpful or very helpful and 62% reported a moderate or major impact of the CL psychology service on their satisfaction with hospitalization (Bullock et al., 2022). Perhaps, even more importantly, 78% of patients reported improved coping skills related to medical illness (Bullock et al., 2022), suggesting that CL psychology services are effective. Measures that assess patient satisfaction and effectiveness of CL psychology services are critical for demonstrating value to patients.

## Conclusion and The Future of CL Psychology

CL psychology remains a growing field with many exciting opportunities to inform the future direction and growth of services. Many healthcare institutions benefit from a CL psychology service and yet to date CL psychology services for the adult population are relatively rare. One of the unique aspects of CL psychologists is the flexibility with which they are able to adapt CL psychology services to offer innovative and effective ways to address emerging contemporary needs of healthcare. CL psychologists possess a unique skill set that is broadly applicable to a wide variety of challenges facing healthcare currently and in the future. The ability to continue to identify applications of CL psychology services to support institutional initiatives and missions will continue to be key in the expansion of the subspecialty. A clear area of growth for the field is the need for additional research to demonstrate the value and effectiveness of CL psychology services and to continue to identify meaningful and measurable outcomes in addition to billing/collections and RVU generation. We believe that strategic collaborations, both internal and external to one’s home institution, are critical for accomplishing these tasks and promoting CL psychology and related research. CL psychologists are charged with conducting research to continue to grow this subspecialty and support the future of CL.

## Data Availability

No datasets were generated or analyzed during the current study.
